# Circadian distribution of epileptiform discharges in epilepsy: Candidate mechanisms of variability

**DOI:** 10.1371/journal.pcbi.1010508

**Published:** 2023-10-05

**Authors:** Isabella Marinelli, Jamie J. Walker, Udaya Seneviratne, Wendyl D’Souza, Mark J. Cook, Clare Anderson, Andrew P. Bagshaw, Stafford L. Lightman, Wessel Woldman, John R. Terry

**Affiliations:** 1 Centre for Systems Modelling and Quantitative Biomedicine, University of Birmingham, Birmingham, United Kingdom; 2 EPSRC Centre for Predictive Modelling in Healthcare, University of Exeter, Exeter, United Kingdom; 3 Department of Neurosciences, Monash Health, Clayton, Australia; 4 Department of Neuroscience, St. Vincent’s Hospital, University of Melbourne, Melbourne, Australia; 5 Department of Medicine, St. Vincent’s Hospital, University of Melbourne, Melbourne, Australia; 6 School of Psychological Sciences and Turner Institute for Brain and Mental Health, Monash University, Clayton, Australia; 7 Centre for Human Brain Health, University of Birmingham, Birmingham, United Kingdom; 8 Bristol Medical School: Translational Health Sciences, University of Bristol, Bristol, United Kingdom; Inselspital, SWITZERLAND

## Abstract

Epilepsy is a serious neurological disorder characterised by a tendency to have recurrent, spontaneous, seizures. Classically, seizures are assumed to occur at random. However, recent research has uncovered underlying rhythms both in seizures and in key signatures of epilepsy—so-called interictal epileptiform activity—with timescales that vary from hours and days through to months. Understanding the physiological mechanisms that determine these rhythmic patterns of epileptiform discharges remains an open question. Many people with epilepsy identify precipitants of their seizures, the most common of which include stress, sleep deprivation and fatigue. To quantify the impact of these physiological factors, we analysed 24-hour EEG recordings from a cohort of 107 people with idiopathic generalized epilepsy. We found two subgroups with distinct distributions of epileptiform discharges: one with highest incidence during sleep and the other during day-time. We interrogated these data using a mathematical model that describes the transitions between background and epileptiform activity in large-scale brain networks. This model was extended to include a time-dependent forcing term, where the excitability of nodes within the network could be modulated by other factors. We calibrated this forcing term using independently-collected human cortisol (the primary stress-responsive hormone characterised by circadian and ultradian patterns of secretion) data and sleep-staged EEG from healthy human participants. We found that either the dynamics of cortisol or sleep stage transition, or a combination of both, could explain most of the observed distributions of epileptiform discharges. Our findings provide conceptual evidence for the existence of underlying physiological drivers of rhythms of epileptiform discharges. These findings should motivate future research to explore these mechanisms in carefully designed experiments using animal models or people with epilepsy.

## Author summary

65 million people have epilepsy worldwide. Many of these people report specific triggers that make their seizures (the primary symptom of epilepsy) more likely. Here, we use a mathematical model to understand the relationship between possible triggers and rhythms in epileptiform activity observed across the day.

The mathematical model describes the activity of connected brain regions, and how the excitability of these regions can change in response to different stimuli. Based on data collected from people with idiopathic generalized epilepsy, we identify transitions between sleep stages and variation in concentration of the stress-hormone cortisol as candidate factors that influence how likely it is for epileptiform activity to occur. By including those factors into the model, we show they can explain most of the daily variability. More broadly, our approach provides a framework for better understanding what factors drive the occurrence of epileptiform activity and offers the potential to suggest experiments that can validate model predictions.

## Introduction

Epilepsy is a common neurological disorder, affecting 65 million people globally [1–3]. The primary symptom of epilepsy—seizures—is believed to occur as a result of disruptions in the level of neuronal excitability. In particular, mechanisms that govern the normal balance between excitation and inhibition can become compromised causing parts of the brain to become hyperexcitable, which can be characterised at different scales. For example, at the cellular level it is strongly associated with the so-called paroxysmal depolarization shift (PDS) of cortical pyramidal cells [4, 5]. At the macroscale, it manifests in pathological electrical activity, captured using electroencephalography (EEG), called epileptiform discharges (EDs). EDs can be thought of as an umbrella term that encompasses both interictal (i.e., between seizures) epileptiform activity (e.g., spikes) as well as ictal activity (i.e., seizures).

Epileptiform activity has classically been thought to occur at random, but recent studies have presented compelling evidence for underlying rhythmicity in EDs [6–11]. Although such cycles have been shown to follow several temporal scales, including ultradian, circadian, multidien and even circannual rhythms [12, 13], relatively little is currently known about the mechanisms—i.e., physiological perturbations—governing these rhythms and how intrinsic and extrinsic factors can modulate the likelihood of EDs. This limits the extent to which this knowledge of rhythmicity can be used for clinical benefit.

Many people with epilepsy identify triggers that appear to make them more likely, and some of these triggers are physiological factors known to influence cortical excitability. The most common of these are stress, sleep, hormones, and medication [14–19].

In this study, we consider some of these factors as candidate mechanisms that modulate the likelihood of EDs, and present a modelling approach to provide insight into the mechanisms underlying observed distributions of EDs [19].

The mammalian stress-response is driven by circulating glucocorticoid hormones: predominantly cortisol in humans and corticosterone in rodents, herein CORT. Ultradian and circadian rhythms of CORT are controlled by the hypothalamic-pituitary-adrenal (HPA) axis, a neuroendocrine axis, wherein a delayed negative-feedback loop mediates hormone secretion from the pituitary and adrenal glands [20]. The impact of CORT on brain function is well established. For example, rapid changes in CORT secretion not only have major effects on glucocorticoid receptor activation in the brain [21] but also major effects on cognition [22]. Furthermore, Karst et al. [23] demonstrated that neuronal excitability is rapidly and reversibly determined by changes in CORT levels. At the macroscale, Schridde et al. [24] observed a CORT dose-dependent increase in EDs in the genetically in-bred Wistar Albino Glaxo/Rij (WAG-Rij) model of human idiopathic generalized epilepsy (IGE). A similar relationship has been found more recently in people with stress-sensitive focal epilepsies [25].

One of the most direct ways of measuring human cortical excitability is via transcranial magnetic stimulation (TMS), with motor and/or EEG responses taken as a proxy for excitability. With this approach, prolonged wakefulness leading to sleep deprivation has been shown to increase excitability or alter the excitatory-inhibitory balance of the supplementary motor cortex [26–28]. In addition, TMS-derived cortical excitability is also modulated by circadian phase, such that excitability is lowest in the early evening prior to bedtime, and peaks at the end of the biological night [29]. These observations have not always been consistent [30] with some suggestion of differences between participants with and without epilepsy [30]. These results are generally consistent with the changing probability of EDs associated with sleep deprivation and/or fluctuations in the circadian rhythm [31, 32]. The probability of EDs also varies through the sleep cycle, with non-rapid eye movement (NREM) sleep generally having a facilitatory effect, and REM sleep an inhibitory effect [32–34]. The latter observation is consistent with the increase in TMS-defined excitability associated with selective REM sleep deprivation [35].

However, the complexity of these interrelating factors, alongside the difficulty of simultaneously measuring their physiological correlates, makes unpacking them challenging. In this paper, we analysed distributions of EDs from 107 people with IGE collected over 24-hours. We found evidence to support the existence of two primary groups with different mechanisms driving the overnight likelihood of EDs and their likelihood during the day. To explore possible contributing factors underpinning these different mechanisms we developed a mathematical modelling framework that:

a) describes transitions between background states and EDs;
b) relates excitability to the likelihood of these transitions;
c) considers the impact of intrinsic and extrinsic factors on excitability.

We calibrated model parameters using independently collected 24-hour hormone profiles from 6 healthy participants, and sleep staged polysomnography data from 42 healthy participants. We used synthetic minority oversampling to account for discrepancies in group size, enabling us to generate synthetic distributions of EDs. We explored the goodness of fit between these model derived distributions and those observed in the cohort of people with IGE. Our mathematical analysis revealed evidence to support the view that the likelihood of EDs is modulated by both transitions in sleep stages, as well as by ultradian fluctuations in cycling CORT levels.

## Results

We analysed distributions of EDs derived from 24-hour EEG recordings from 107 subjects with IGE (see Materials and methods for a detailed description of this data-set).

### Variability in the circadian distribution of epileptiform discharges

**Fig 1:**
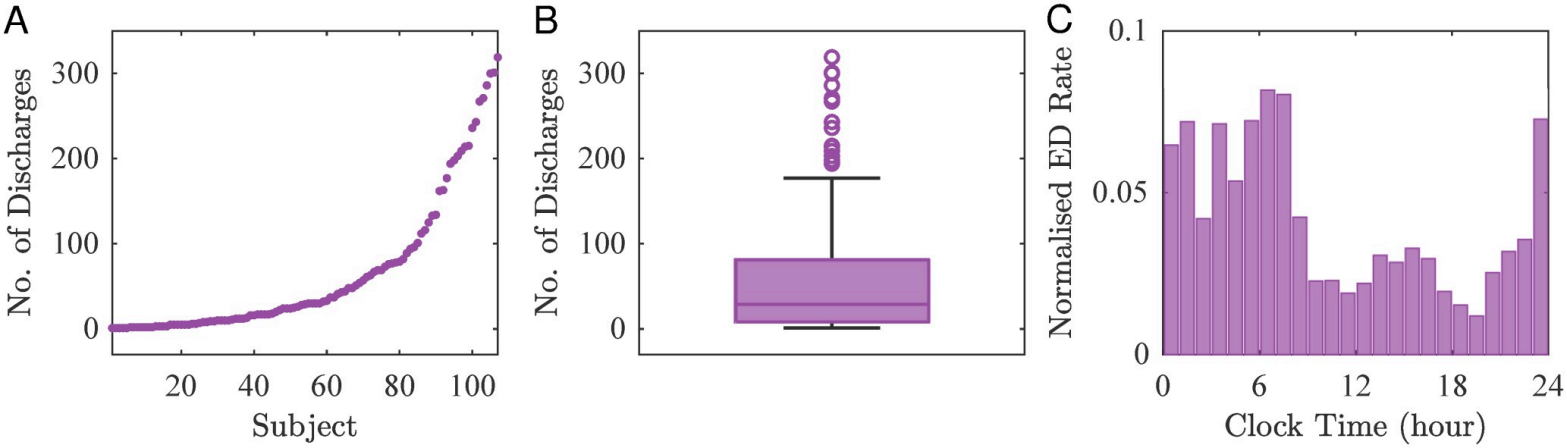
ED distribution in people with IGE. (A) Number of EDs from 107 subjects with idiopathic generalized epilepsy (IGE). (B) Boxplot shows basic sample statistics (minimum, lower quartile, median, upper quartile and maximum) of the number of EDs. (C) Normalised EDs rate per hour.

We found that the median number of EDs over 24 hours was approximately 29, although several individuals had more than 200 events (Fig 1A and 1B). Examination of normalised ED patterns on an hourly basis (i.e., for each individual, the number of EDs at each hour was divided by their total number of EDs and we then normalised over the cohort), suggested that the likelihood of EDs varied across the day (Fig 1C).

**Fig 2:**
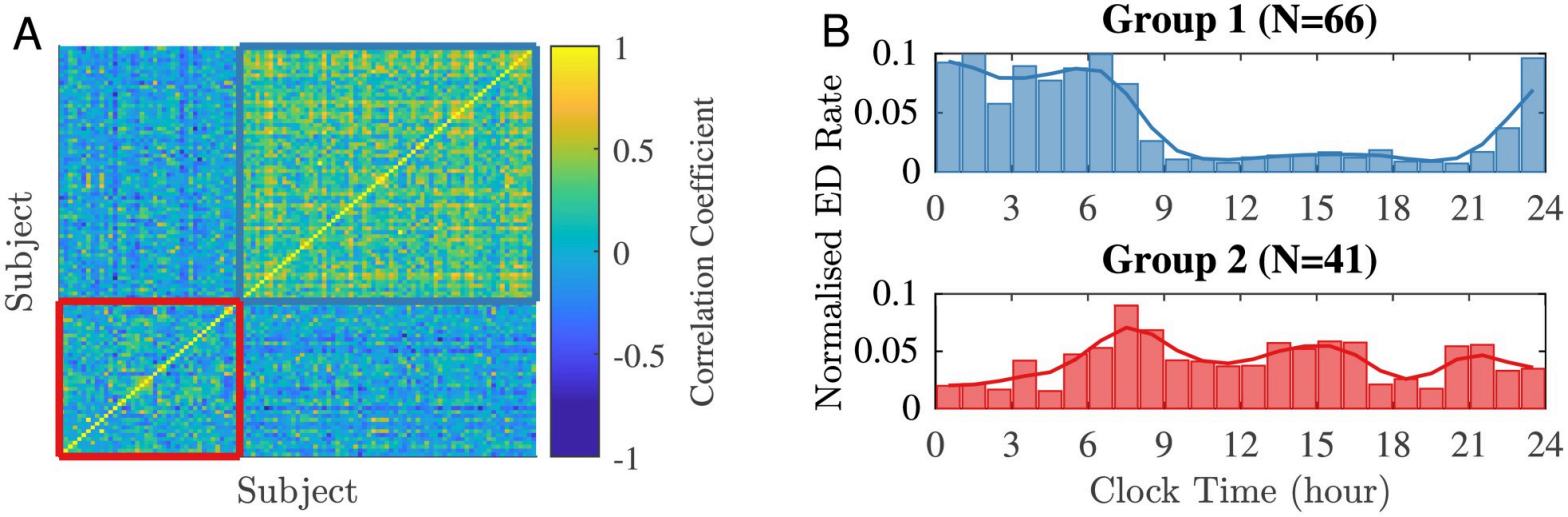
IGE subjects organised based on different circadian ED distribution patterns. (A) A pairwise cross-correlation matrix (of size 107 × 107) was calculated using ED hourly rate patterns in order to establish similarities within the IGE cohort. (B) Group 1 (blue, N = 66) and Group 2 (red, N = 41) were identified based on the similarities of hourly ED rate.

To investigate the possible temporal distribution of EDs across the 24-hour day (herein referred to as the ‘circadian distribution’), we first considered similarities between subjects. We used MATLAB R2021a (MathWorks Inc., Natick, MA) to compute the cross-correlation coefficients of time series representing the individual hourly ED rate. This leads to a correlation matrix *C*, with entries *C*_*ij*_ corresponding to the similarity between the pattern of EDs in subject *i* and in subject *j* (Fig 2A). The closer the value of *C*_*ij*_ to 1, the more similar the distribution of EDs of subject *i* and subject *j*. Subsequently, we clustered subjects according to their correlation coefficients using *k*-means clustering [36] and the Calinski-Harabasz criterion [37] to optimise the number of clusters (see Fig A in S1 Text). This analysis revealed two primary groups within the overall cohort of people with IGE that displayed different temporal ED distribution patterns: Group 1 of 66 individuals and Group 2 of 41 individuals (Fig 2B). Importantly, the identified clusters were found to be consistent across a range of bin widths (45–90 minutes) and when the time series were aligned to sleep times (see S1 Text).

We found that the groups identified by our cluster analysis were not caused by imbalances in the type of epilepsy. Specifically, individuals with IGE were classified into childhood absence epilepsy (CAE), juvenile absence epilepsy (JAE), juvenile myoclonic epilepsy (JME), generalized epilepsy with generalized tonic-clonic seizures only (GTCSO), and genetic generalized epilepsy unspecified (GGEU) according to the criteria published by the ILAE [38]. We fitted a linear model (see Table B in S1 Text for details) to assess the dependence of the groups on epilepsy type, finding no evidence of an association (*p* = 0.756).

### Candidate mechanisms impacting the distributions: Sleep and CORT

We explored candidate mechanisms that could explain differences in ED distributions between the two groups identified by our cluster analysis. The (empirical) likelihood of EDs in Group 1 (Fig 2B top) displayed a significant increase in the propensity for EDs during the night and lower levels during day-time. In contrast, the likelihood of EDs in Group 2 (Fig 2B bottom) displayed greater variation during waking hours.

**Fig 3:**
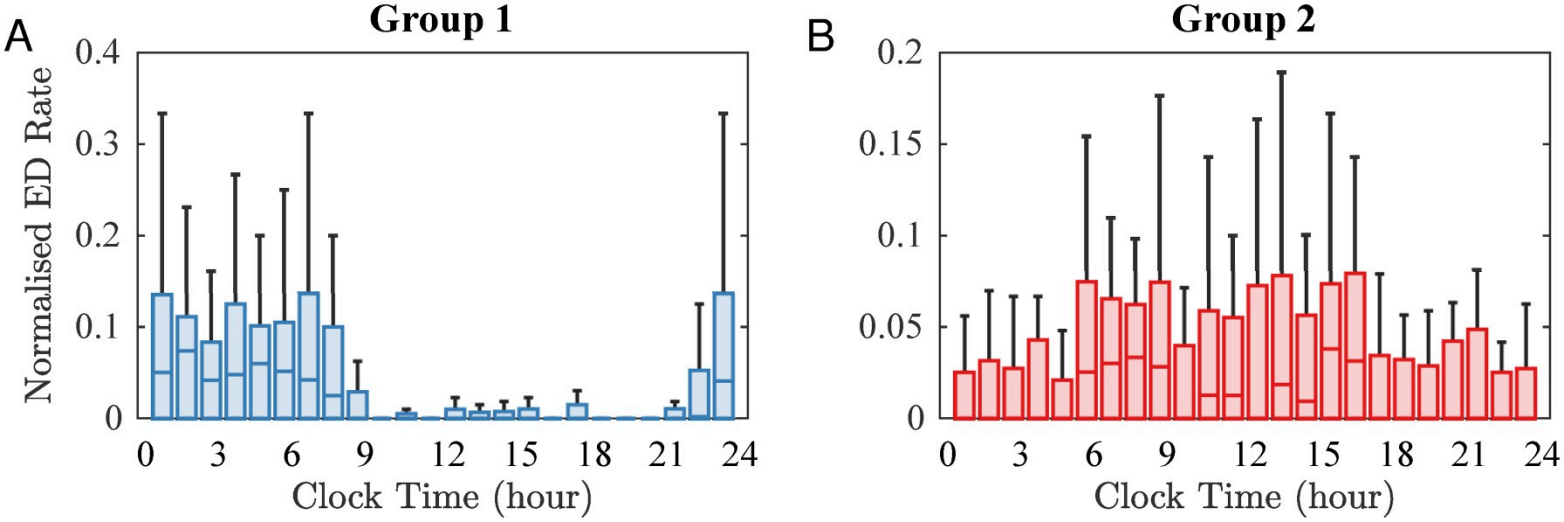
ED occurrence. Boxplots showing the distribution of ED across 24 hours for Group 1 (A) and Group 2 (B). Within each box plot, the central line represents the median, and the bottom and top edges represent the 0.25 and 0.75 quantiles, respectively. The whiskers extend to the most extreme data points not considered outliers.

Fig 3 illustrates the variability of ED distribution across 24 hours for the individuals in Group 1 (Panel A) and Group 2 (Panel B).

We employed a mixed-effects Poisson regression model (see Materials and methods and Table C in S1 Text for more details) to study the temporal distribution of epileptiform discharges and the impact of sleep independently in both groups:

$$\begin{array}{c} \text{ED}∼\text{Time}+\text{Sleep}+(1|\text{Subject}) \end{array}$$ (1)

where the observed variable ED corresponds to the ED occurrence during the 24-hour time window in either Group 1 or Group 2, the predictor Time represents the circadian time (hours), and Sleep indicates whether the individual is sleeping or not. Due to the intra-subject variability, we introduce the variable Subject as a random factor.

In both groups, there is a statistically significant change in ED counts across time blocks and sleep (*p*-value <0.001). This suggests an impact of sleep on the ED occurrence in Group 1 as well as in Group 2. This result can be explained by observing that the morning peak in ED events recorded in Group 2 starts during sleep time.

**Fig 4:**
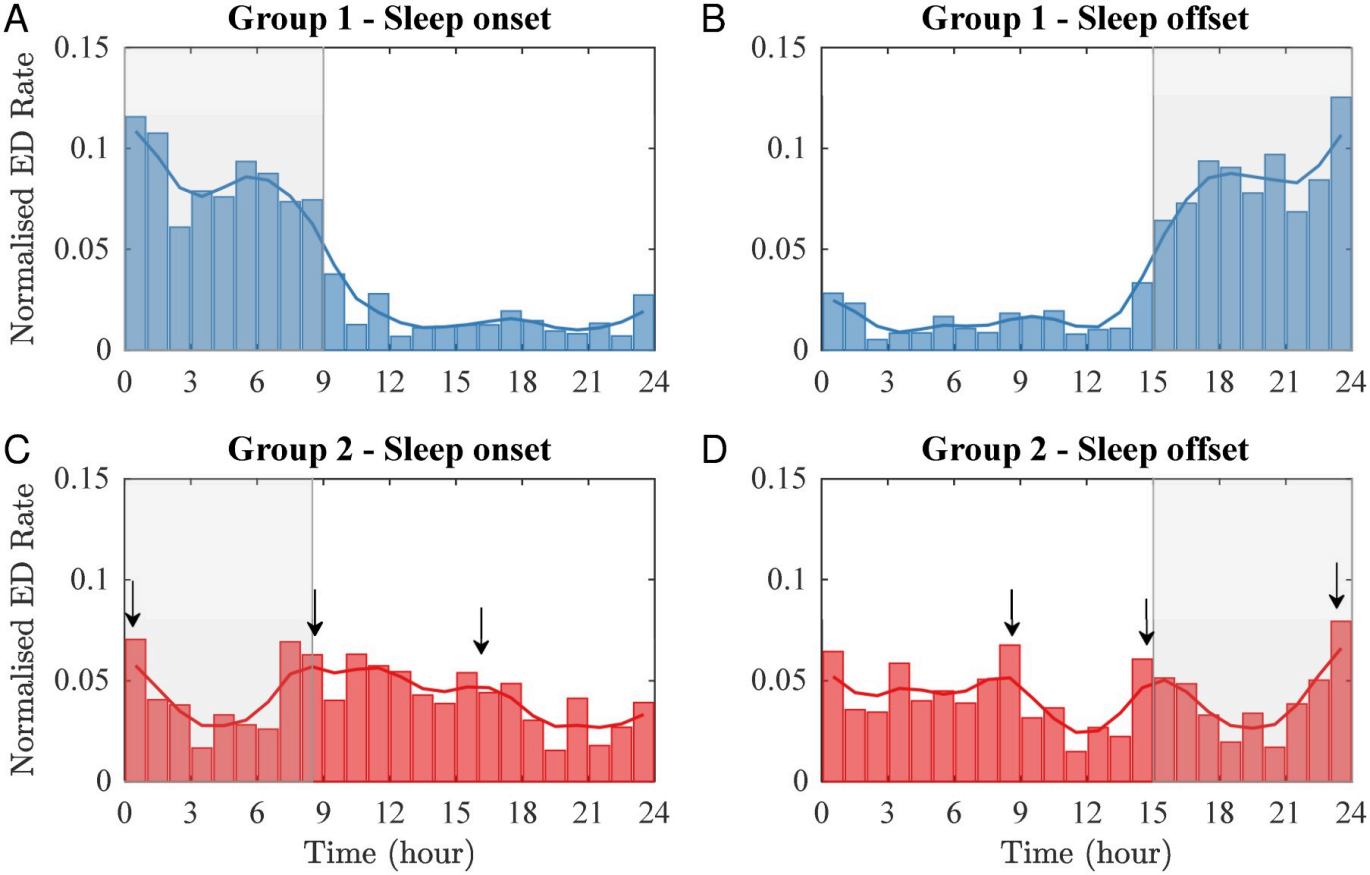
Impact of timing of sleep and its duration on ED distributions. Epileptiform discharges for Group 1 (top row) and Group 2 (bottom row) with time normalised such that *t* = 0 corresponds with sleep onset (A and C) and with sleep offset (B and D). The transparent grey box highlights the average habitual sleep period. The black arrows indicate the peaks in the ED distribution in Group 2. The peaks were determined by identifying the local maxima of the density function (solid red line).

To further assess the impact of inter-individual timing of sleep and its duration on ED distributions, we adjusted time within each subject such that *t* = 0 corresponded to either their sleep onset or sleep offset. The resulting distributions are presented in Fig 4A–4D. For Group 1, we found that the ED rate was higher for approximately 9 hours starting at habitual sleep onset (Panel A), while it was relatively low during the rest of the day. In Panel B, we observed the same trend but shifted to the 9 hours before waking. For Group 2 (Fig 4C and 4D) we did not find increased levels of EDs during sleep; instead, the distribution suggests a potential daytime ultradian rhythm.

To quantify this more explicitly, we introduced the parameter *F*_*i*_, *i* = 1, 2 to measure the fraction of EDs occurring during sleep for each group:

$$\begin{array}{c} F_{i}=\frac{1}{N_{i}}\sum_{j=1}^{N_{i}}\frac{ED_{S,j}}{ED_{\text{tot},j}}. \end{array}$$ (2)

Here *N*_*i*_ is the number of subjects in Group *i*, *ED*_S,*j*_ and *ED*_tot,*j*_ are the numbers of ED occurrences for the *j*^*th*^ subject in the *i*^*th*^ group occurring during the individual’s sleep time and across the full 24-hour period, respectively. We found *F*_1_ = 0.8, suggesting that 80% of EDs in Group 1 were clustered during the sleep period. In contrast, *F*_2_ is 0.37, suggesting that in Group 2 just over a third of discharges occur during the sleep period, consistent with the 8–9 hour sleep time (i.e., a third of 24-hour). Interestingly, for Group 2 we found three peaks of similar height around 8 hours prior to sleep, sleep onset, and sleep offset (Fig 4C). We found the equivalent pattern when aligning by sleep offset (Fig 4D). A similar pattern can be observed in the levels of plasma CORT over 24-hours, which displays a circadian rhythm that reaches a peak soon after awakening and a nadir during the night [39, 40].

### Mathematical modelling and the relationship between sleep, CORT and the distribution of EDs

To explore the hypothesis that sleep and CORT impact the distribution of EDs, we used a computational modelling framework that employed a network model as a natural choice to simulate the behaviour of interconnected brain regions that could potentially influence each other. Within this framework, we assessed how changes to the overall excitability of brain regions impacted the overall likelihood of *in silico* EDs. The model simulates transitions between a background state and an epileptiform state in a network, and how excitability of nodes—the brain regions—can be influenced by external perturbations (see Materials and methods for a detailed description of the model). In our study, we designed a 4-node network to simulate the four regions of the brain and their connections. Our choice was to provide a phenomenological example of brain activity and to reduce the computational demands. Results were similar when smaller (*N* = 2, 3) or bigger (*N* = 5) networks with different node degrees were considered (see Fig D in S1 Text).

**Fig 5:**
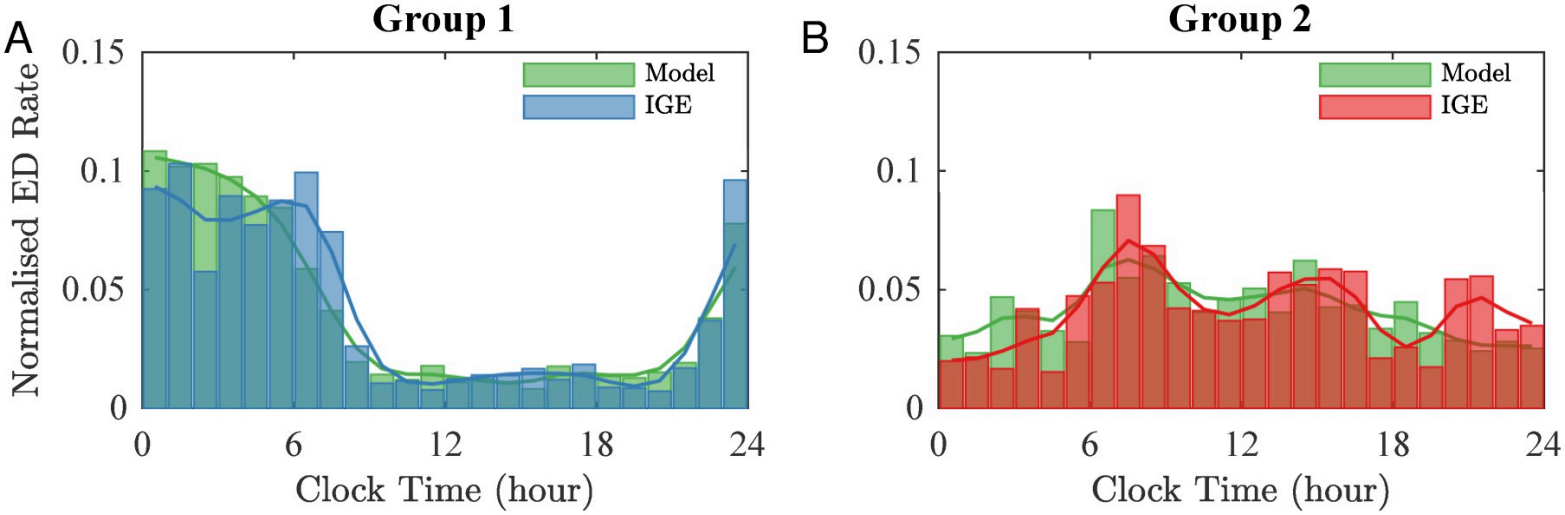
Model results compared with IGE data. (A) Histogram of EDs from Group 1 with IGE (blue) and histogram of EDs simulated using the model with λ_ext_ defined to mimic the different brain excitability during sleep stages (green). (B) Histogram of EDs from Group 2 with IGE (red) and histogram of EDs simulated using the model with λ_ext_ defined to mimic the impact of CORT on the brain excitability (green).

For Group 1, we used sleep-staged polysomnography data collected from healthy controls (see Materials and methods) as an external input to the excitability of the model λ_ext_(*t*). In Fig 5A we compared the model output for a virtual cohort (i.e., a set of synthetic time-series generated from the observed data) of 66 individuals (in green) with the observed ED distributions for Group 1 (in blue).

The model predicted a sharp increase in ED occurring during the first part of the sleep period, followed by a sharp decrease in the morning. The slow reduction in the number of EDs during the night is consistent with the observation that NREM sleep is predominant during the first part of the sleep, while REM is predominant during the second half [41]. Although the model captured most of the Group 1 ED variability (*R*^2^=0.9), it failed to capture the bimodal distribution in ED rate shown in the overnight data. It further failed to capture daytime variability in the ED rate, suggesting the presence of at least a second mechanism governing the ED propensity.

For Group 2, we used levels of CORT measured from healthy controls over the course of 24 hours (see Materials and methods) as an external input to the excitability of the model λ_ext_(*t*). The model prediction for a virtual cohort of 41 individuals is shown in Fig 5 (in green). Comparing the model results with the data (in red), we found that the model captures the morning and afternoon peaks displayed by Group 2, although the latter occurs about an hour earlier in the model. We also note that the simulation does not account for the evening peak around 21:00. The overall variability explained by CORT in Group 2 is ∼60% (*R*^2^=0.59).

To verify that the external factors do have an impact on the ED distributions simulated by our model, we introduced the concept of ‘null model’ as the model with no external input, i.e., λ_ext_(*t*) = 0. A linear model was fit to investigate the impact of time over the ED occurrence simulated with the ‘null model’ and the model with the external perturbations (sleep and CORT). In both groups, there is a statistically relevant change during the day (*p* <0.001) when either sleep or CORT are simulated, while there are no changes in the null model (*p* >0.1). See Tables D and E in S1 Text for the details of the statistical analysis. Moreover, we performed simulations when CORT and sleep are the only mechanisms in Group 1 and 2, respectively. In both cases, *R*^2^ is negative, indicating that the model performs worse than the fixed mean value (see Fig E in S1 Text).

### Combined mechanism: Sleep and CORT

**Fig 6:**
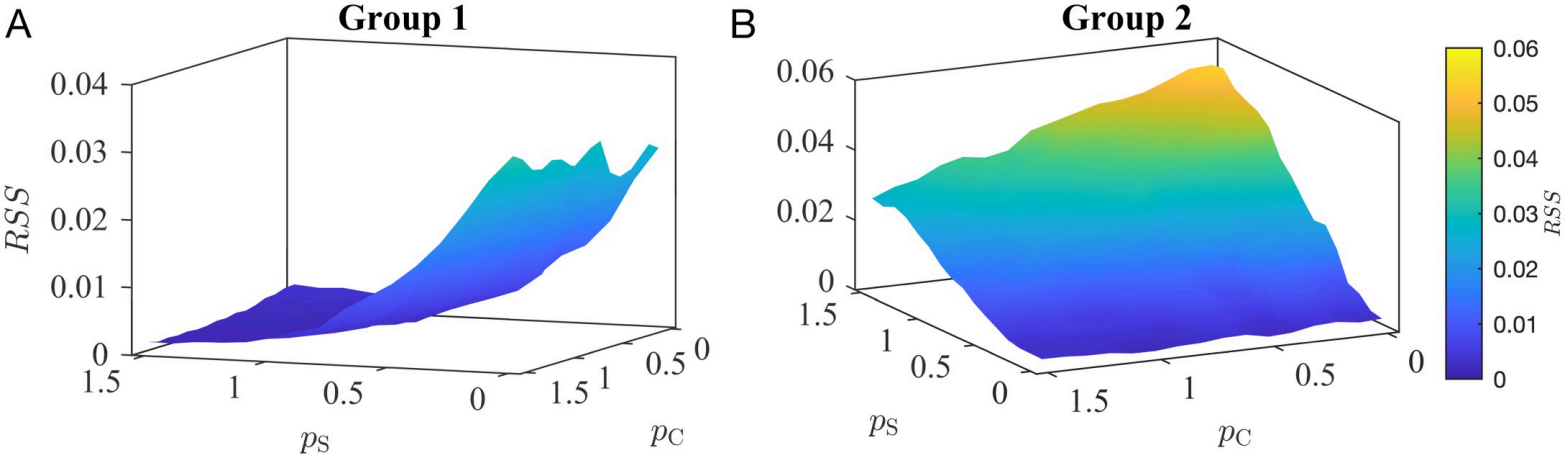
*RSS* values for the combined mechanism. Values of the residual sum of squares (*RSS*) computed over a grid of values of *p*_S_ and *p*_C_ for Group 1 (A) and Group 2 (B).

For each group, we identified candidate mechanisms that could explain the majority of the observed distribution of EDs. However, we found that the model failed to capture some variability. For example, in Group 1 the bimodal distribution during sleep, as well as some variability during the day, was not fully explained by the model. We therefore explored how combining the mechanisms of sleep and CORT impacted the ED distribution. The excitability is driven by a linear combination of sleep and CORT levels, with the strength of the influence of each factor defined by parameters *p*_S_ and *p*_C_, respectively (see Materials and methods). Each parameter can vary from 0 (no impact on ED occurrence) to 1.5 (strong impact on ED occurrence). Both parameters are considered only to be positive as both sleep and CORT have been shown to increase levels of brain excitability, whereas negative values would instead reduce brain activity. We used residual sum of squares (*RSS*) to assess the overall fit (see Fig 6 and Fig F in S1 Text for *R*^2^). We also implemented the maximum-likelihood estimation (MLE) method and the Metropolis-Hastings Monte Carlo Markov Chain (MCMC) to estimate the parameters *p*_S_ and *p*_C_ and found consistent results (see S1 Text).

**Fig 7:**
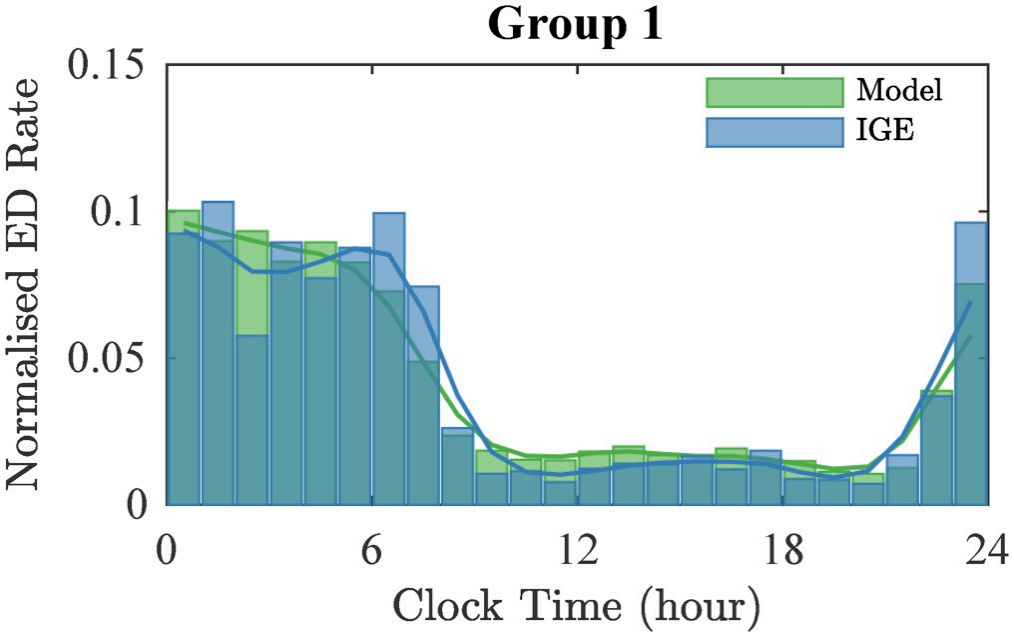
Best model fit for Group 1 compared with IGE data. Histogram of EDs from Group 1 with IGE (blue) and histogram of EDs simulated using the model with λ_ext_ defined to mimic the impact of the combined mechanism (sleep and CORT) on excitability (green). In this simulation, *p*_S_ = 1.1 and *p*_C_ = 0.6.

In Group 1, we found the best fit (lowest *RSS* values) was obtained when *p*_S_, *p*_C_ > 0 (Fig 6A). This result is consistent with our previous observation that sleep can explain the overnight peaks in EDs, with the contribution of CORT explaining variability during the day. This result suggests the coexistence of the two mechanisms (sleep and CORT) in Group 1. Fig 7 shows the model output corresponding to the lowest *RSS* for this group, which is when both sleep and CORT terms are present with *p*_S_ = 1.1 and *p*_C_ = 0.6. Combining these mechanisms increases the explained variability from 90% (only sleep) to 95% (*R*^2^ = 0.95).

Conversely, the lowest values of *RSS* in Group 2 were obtained when *p*_S_ = 0 (Fig 6B), suggesting that the best fit is obtained when CORT is the sole mechanism considered in the model (as in Fig 5B) (*R*^2^ = 0.59). Although additional mechanisms could be considered in explaining the remaining variance using this computational framework it is important to recognize the relatively modest sample-size of the remaining subgroups combined with the possibility of true random events (see Figs I and J and K in S1 Text).

## Discussion

The aim of this study was to provide a computational framework for assessing how the likelihood of epileptiform discharges is impacted by different physiological mechanisms and processes, such as sleep and changes in concentration of the stress-hormone CORT. First, a data-driven analysis of the distributions of epileptiform activity from a large cohort of people with generalized epilepsies revealed the presence of two distinct groups within this cohort. To explain the underlying differences between these groups, we used a phenomenological mathematical model for simulating the activity of brain networks and excitability. Using this framework, we found that the patterns in the first group (Group 1) are strongly correlated with sleep, whereas the daily changes in ED likelihood in the second group (Group 2) can partially be explained by CORT. This framework provides an intuitive way of assessing the impact of external factors (e.g. sleep, stress, medication, hormonal fluctuations) on the overall likelihood of epileptiform activity, and can be used in the context of future experimental studies.

A data-driven approach was applied to the histograms of epileptiform discharges derived from 107 subjects with generalized epilepsies. First, we found that correlation and cluster analysis suggested the presence of two distinct groups within the overall cohort (of size 66 and 41 respectively). These two groups were not aligned with the clinical sub-types of IGE. Determining the periods of maximum ED likelihood in the two groups suggested sleep stages and CORT levels as candidate drivers for these ED distributions. EDs are increasingly understood as emerging from brain networks, with alterations to both the connectivity between brain regions, as well as the dynamics within regions, contributing to this emergence [42, 43]. In this regard, both sleep stage and levels of CORT have been shown to impact both functional connectivity [44, 45] and cortical excitability [46, 47]. Several studies have shown the correlation between sleep and epileptiform discharges [32, 34, 41] and how vigilance states may influence the likelihood of EDs in subjects with IGE [46, 48, 49]. CORT is the main stress hormone in humans and its production and secretion are controlled by the hypothalamic-pituitary-adrenal (HPA) axis, the primary stress response system [20]. In stressful situations, the activity of the HPA axis increases, resulting in a higher secretion of CORT. In unstressed, basal conditions, cycling levels of CORT rise and fall over the day, with characteristic ultradian pulses [50]. This finding is consistent with the literature and self-reported data showing ED frequency increasing during the night time, early in the morning, and in stressful situations.

To investigate the impact of sleep and CORT on the ED likelihood during the day, we employed a phenomenological mathematical model to simulate brain excitability when perturbed by those external forces. Unlike in previous works where the variation of the brain excitability was constant [51] or perturbed by a fixed constant [52], this model describes cortical excitability as a dynamical variable that is modulated by dynamic external factors, such as sleep or CORT. We used sleep stages and CORT levels collected from healthy subjects to inform the dynamics of the variable representing the status of brain activity. Although from different cohorts, the circadian patterns of the hormone are robust across individuals [53]. However, in future work, the analysis should include CORT levels and sleep stage data derived from the EEG from the same individual, given that both of these variables show considerable inter-individual variability. Despite this limitation, our work shows a good fit between our model simulations and the observations. Indeed, we find that sleep accounts for 90% of the variability in Group 1 (*R*^2^ = 0.9) and CORT for ∼60% (*R*^2^ = 0.59) in Group 2.

Importantly, sleep alone cannot account for the changes in ED likelihood during wakefulness observed in Group 1. Furthermore, the model predicts a reduction in ED likelihood during the sleep time after an initial sharp increase during the first hours. This effect can be explained by the fact that NREM sleep, which is positively correlated to an increase of EDs, is predominant during the first third of the sleep period. However, the data shows an increase in ED occurrence before waking, which the model simulations fail to capture. Given that the level of CORT is known to increase around waking, this result suggests a combined effect of sleep and CORT. This result is quantitatively highlighted by the improvement in the accuracy of our model prediction when a combination of sleep and CORT have been considered and by the high percentage of variability explained by the combined model (95%, *R*^2^ = 0.95). It is important to emphasise that we only considered linear combinations, and future work could investigate a richer class of non-linear interactions and effects, especially given that sleep and CORT themselves interact. This interaction may potentially lead to non-linear impacts on the likelihood of EDs.

Our model predicts peaks occurring during the day, for example, one around 13:00 and one around 19:00, in Group 2. Those two peaks seem to occur a couple of hours earlier in the model than in the IGE cohort. The reason for such behaviour requires further investigation. One explanation could be additional physiological or behavioural drivers that we have not yet accounted for. Alternatively, it is important to highlight that CORT levels were measured in an independent control cohort. A future study would critically include simultaneous recordings of EEG and CORT, as well as detailed summaries of any anti-seizure treatment (e.g. timing and dose). Finally, it is important to consider the presence of inherent natural variability in these types of distributions in future studies.

In summary, we provide a mathematical model as a tool to examine the role of external factors on the modulation of ED likelihood. We provide quantitative evidence that underlying physiological modulators for ED events exist. We identified sleep and CORT as such modulators by comparing our model predictions with data on ED events collected from IGE patients. Our choice of such factors is guided by the ED distribution in the EEG data and by previous studies investigating sleep and CORT, and the influence they have over the cortical excitability dynamics. Using only these two processes, we are able to account for the majority of the variability in the two groups. However, our results are not technically inferential due to the different data sources, nor do they exclude other potential mechanisms affecting cortical excitability during the day, such as sleep deprivation or anti-seizure medication [29, 31]. Furthermore, other factors showing circadian rhythms, such as melatonin production or glucose levels, have also been shown to impact seizure incidence [54, 55, 55, 56]. Further research is needed to fully understand the overall mechanism underlying the modulation of ED events. In particular, simultaneous recordings of EEG and those factors are necessary to overcome the high intra- and inter-individual variability of the latter. Critically, measurements should be taken from the same individual over prolonged periods, which would then inform the model framework (in particular the network structures and the excitability dynamics) with robust statistical testing (e.g., null distribution).

Ultimately, the modelling approach provides a starting point to better understand what drives the occurrence of epilepsy-related activity observed in recordings of the brain.

## Materials and methods

### Ethics statement

The EEG study was approved by the Human Research Ethics Committees of St. Vincent’s Hospital and Monash Health. A written informed consent was obtained from all participants included in the study. See [57] for more details on the data collection and processing. The sleep study was approved by the Monash University Human Research Ethics Committee (CF14/2790-2014001546; 2017-4204-11012; 2017-6008-8120; and 2020-5453-43401).

### Statistical analysis

Statistical analysis was done using MATLAB R2021a (MathWorks Inc., Natick, MA). A linear model was fit using the MATLAB command fitlm to assess the dependence of the groups on epilepsy type with the formula Group∼Syndrome, where the predictor Group corresponds to the group the individual has been assigned to and Syndrome to the individual’s epilepsy type, as described in Results and Table B in S1 Text. We also employed the function fitlm to fit a linear model to assess whether the model outputs are only due to random noise using the formula Y∼Time+Sleep, where *Y* is either the null model (no external perturbation) or the model simulation with sleep and CORT only for Group 1 and 2, respectively, Time represents the circadian time (hours), and Sleep indicate whether the individual was sleeping or awake (see Results and Tables D and E in S1 Text).

A linear mixed-effects Poisson regression model was fit using the MATLAB command fitlme to assess the dependence of the ED distribution on Time and Sleep. The model formula is as follows: ED∼Time+Sleep+(1|Subject), where the predictor ED corresponds to the different ED occurrence across the 24 hours and the variable Subject is introduced as a random factor to account for the intra-subject variability, as described in Results and Table C in S1 Text. Similarly, we fit a mixed-effects Poisson regression model using the command fitlme to study the differences in cortisol levels across the subjects with the formula CORT∼Subject+(1|Time), where CORT corresponds to the CORT levels for each individual (Subject), and Time is the random factor to account for the physiological daily changes of the hormone (see Table G and Fig L in S1 Text).

### EEG data: Epileptiform discharges in people with idiopathic generalized epilepsy

EDs were identified by an experienced EEG reader (U.S.) within EEGs from 107 people diagnosed with idiopathic generalized epilepsy (IGE). Scalp EEG recordings were collected for 24 hours using a 32-channel ambulatory EEG system (Compumedics Ltd.; Melbourne, Australia). Gold cup electrodes were attached with electrode paste according to the international 10–20 system. Subjects were encouraged to have at least seven to eight hours of night-time sleep prior to the EEG recording to guarantee optimum capture of ED.

### EEG data: Sleep-stages from healthy participants

Sleep-stages from 77 healthy participants were identified from EEG data collected at Monash University (Melbourne, Australia). Sleep polysomnography (PSG) was recorded across two consecutive nights in the laboratory (Compumedics Grael, Melbourne, Australia), using a bi-lateral 18-channel EEG, and two electro-oculographic (EOG, left and right outer canthi) and three electro-myographic (EMG, sub-mentalis) channels. EEG data were sampled at 512Hz. Sleep data for night 2 (following adaptation to the laboratory on night 1), were scored by a trained scorer, and in accordance with AASM criteria [58]. We restrict our analysis to the 42 participants with sleep efficiency equal to or higher than 85% for night 2 (Table 1), as values less than this can be indicative of sleep disturbance.

**Table 1:** Characteristics of the subjects from the sleep cohort used in the simulations.

Number	Female	Male	Mean Age (min, max) [year]
42	8	34	30.71 (18,64)

### Blood data: CORT levels in healthy participants

**Fig 8:**
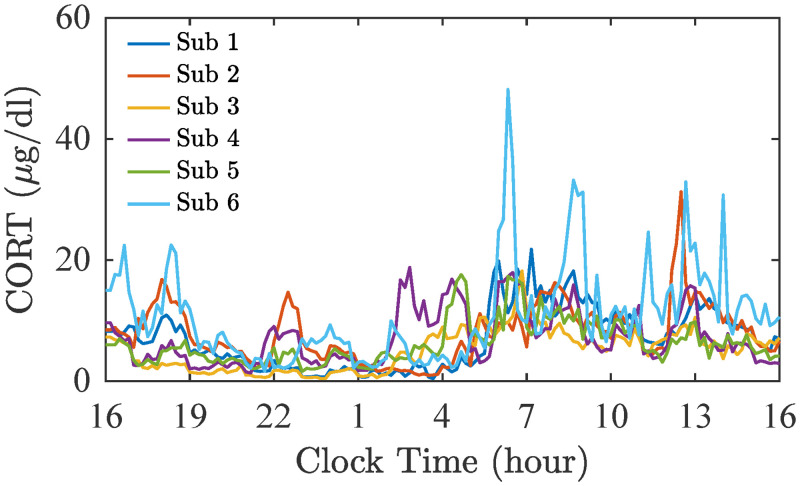
CORT 24-hour recordings. Blood samples for cortisol assay were collected from 6 healthy adult subjects via an intravenous catheter at 10-minute intervals over a 24-hour period.

Cortisol data was kindly provided by Elizabeth A. Young, University of Michigan. Blood samples for cortisol assay were collected from 6 healthy adult subjects via an intravenous catheter at 10-minute intervals over a 24-hour period (Fig 8), as described previously [40, 59].

A mixed-effect Poisson regression model was implemented to investigate the differences in cortisol levels across the subjects. We found that there is a statistically significant inter-subject variability (*p* < 0.001). See Table G in S1 Text for the complete analysis.

### Constructing a virtual cohort

A ‘virtual cohort’ approach was used to compensate for the differences in size and data modality across study groups (Group 1 (people with IGE): 66, Group 2 (people with IGE): 41, CORT (healthy participants): 6, sleep (healthy participants): 42). In order to assess the potential impact of CORT and sleep on the distributions of EDs, new time series were sampled from the sleep and CORT data.

#### Sleep

To compensate for the smaller number of subjects in Group 1 compared to the sleep cohort, we randomly added 24 subjects (without repetition) from the sleep cohort and added them to the original sleep cohort to reach the 66 individuals of this group. On the contrary, Group 2 includes 41 individuals, a smaller group than the sleep cohort. Therefore, a subgroup of the same number of subjects (41) was randomly chosen from the 42 sleep participants.

#### CORT

**Fig 9:**
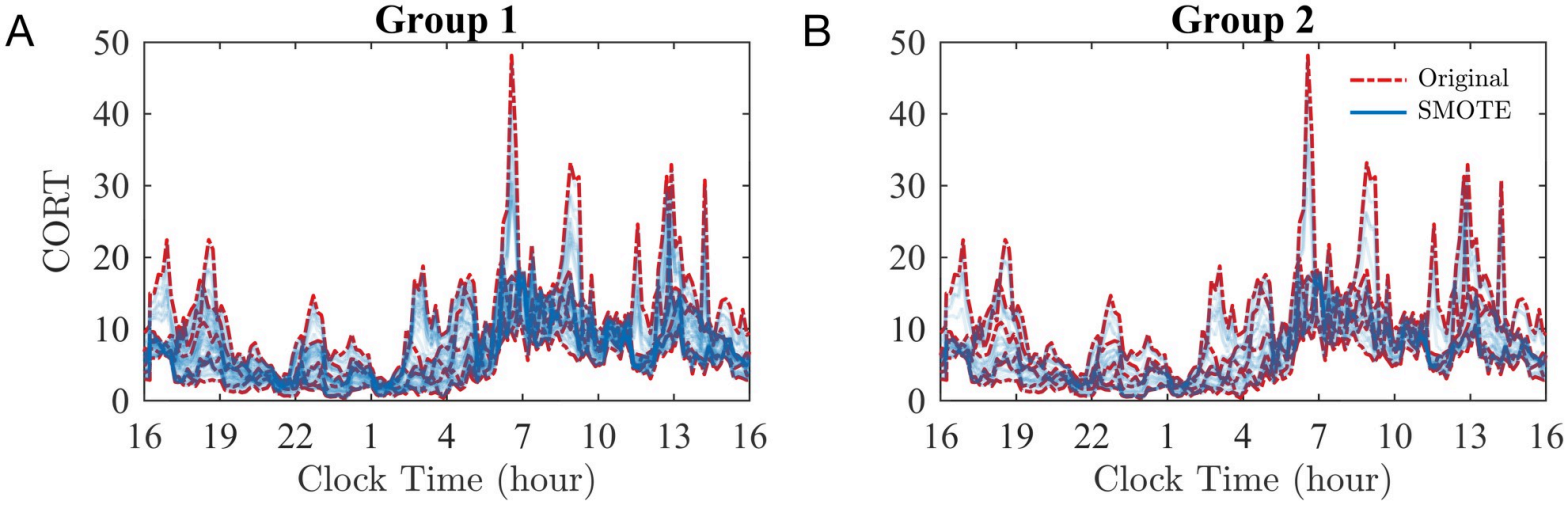
Synthetic CORT surrogates created using SMOTE. Original (red) and synthetic (blue) CORT profiles for Group 1 (A) and Group 2 (B). The synthetic data are obtained with the SMOTE oversampling algorithm with *k* = 3.

To address the significant difference in group sizes (6 healthy participants vs 66 or 41 people with IGE), at each time-point (*i* = 1, …, 145), we used the Synthetic Minority Oversampling Technique (SMOTE) [60] to perform data augmentation. We therefore generated 60 synthetic CORT profiles for Group 1 and 35 synthetic CORT profiles for Group 2 (Fig 9). SMOTE oversampling, based on the *k*-nearest neighbour algorithm, was performed with *k* = 3 (50% of the total).

### Mathematical model

**Fig 10:**
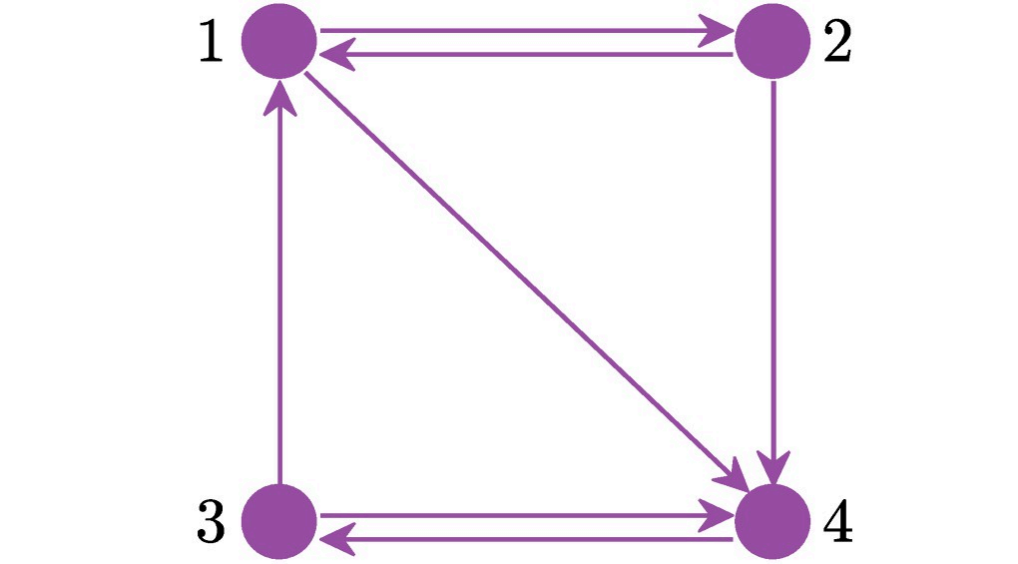
Schematic of the network used in the simulations. The network employed in the simulations is a directed and connected graph.

The model used in this study is based on the normal form of a subcritical Hopf bifurcation [51, 52, 61], whose co-existing states reflect two distinct types of neural activity. The first is a background state, represented by a steady-state solution in the model, whilst the second is an epileptiform state, represented by a high-amplitude oscillation. Transitions between these states are typically governed by either a white noise process or external perturbations. The model equations are given by:

$$\begin{array}{c} \dot{z_{i}}=\left(\lambda_{i}-1+i\omega \right)z_{i}+2z_{i}|z_{i}{|}^{2}-z_{i}{\left|z_{i}\right|}^{4}+\beta \;\sum_{j=1}^{N}A_{ij}\left(z_{j}-z_{i}\right)+\alpha dW\left(t\right)\;, \end{array}$$ (3)

$$\begin{array}{c} \dot{\lambda_{i}}=\frac{1}{\tau }[\lambda_{\text{base}}+{\left(\lambda_{\text{ext}}\right)}_{i}-\lambda_{i}-{\left|z_{i}\right|}^{2}]\;, \end{array}$$ (4)

where *z*_*i*_(*t*) is dynamics of the *i*^*th*^ node (with *i* = 1, …, *N*), *W*(*t*) is a complex Wiener process, λ(*t*) is the excitability of node *i*, λ_base_ the baseline level of excitability, and λ_ext_(*t*) the external perturbations to the excitability. Typical parameter values for the model are given in Table 2, whilst *A* is an adjacency matrix, i.e. *A*_*i*,*j*_ is 1 if there is a connection between the *i*^*th*^ and *j*^*th*^ regions and 0 otherwise. For simplicity, all simulations were performed with a directed and connected 4-node graph (*N* = 4) (Fig 10), in line with [43]. Numerical simulations were obtained using an Euler-Maruyama scheme to find approximate solutions to the system of stochastic differential equations (SDEs) with *dt* = 10^−3^. The method was implemented in MATLAB R2021a (MathWorks Inc., Natick, MA) to simulate 24-hour brain activity (see Fig M in S1 Text) for a representative model simulation.

**Table 2:** Parameters for the mathematical model.

Parameter		
*ω* = 20 rad/s	*β* = 0.35	*α* = 0.055
*τ* = 3 s	λ_base_ = 0.65	

#### Influence of sleep

ED frequency has been observed to vary during sleep and to be higher during non-rapid eye movement (NREM), especially during stages N2 and N3, sleep which is associated with maximal synchronization, than during rapid eye movement (REM) sleep [62].

Therefore, we initially set λ_ext,sleep_ to its maximum value during the NREM state and to 0 during the REM phase. More precisely, λ_ext,sleep_ = 1 during N2 and N3, λ_ext,sleep_ = 0.5 during N1 and λ_ext,sleep_ = 0 during REM and wakefulness. We then rescaled λ_ext,sleep_ by the factor *r*_S_ = 0.11 that was estimated to minimize the *RSS* when sleep is considered the only external factor in Group 1.

**Fig 11:**
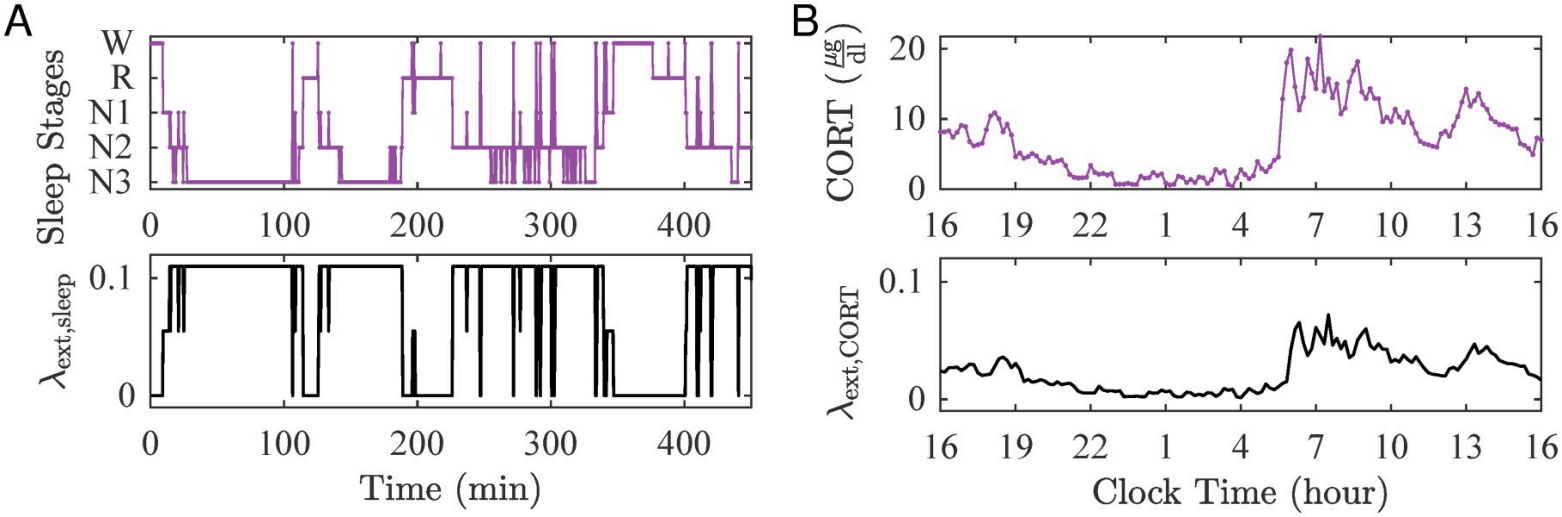
Modelling external perturbations informed by data. The external perturbation to brain excitability due to sleep, λ_ext,sleep_, and CORT, λ_ext,CORT_, were informed by using sleep stages (A) and CORT levels (B), respectively.

Panel A in Fig 11 illustrates a hypnogram representing the sleep stages recorded from a representative control participant (purple, top) and the corresponding λ_ext,sleep_ (black, bottom).

#### Influence of CORT

We modelled λ_ext,CORT_ based on the concentration values of CORT. Also, we account for the delay due to the non-genetic effect of CORT [63], by introducing a delay *ϕ* (min) in the λ_ext,CORT_ compared to the corresponding CORT profile. In our simulations, *ϕ* is from a normal distribution N(13,5) [64, 65]. We then rescaled λ_ext,CORT_ by a factor *r*_C_ that was found by optimising the simulated ED rate, when CORT is considered as the only external factor, and the data from Group 1. By minimising *RSS* we found the rescaling factor for CORT data to be *r*_C_ = 0.0033.

Panel B in Fig 11 illustrates the CORT profiles (purple, top) and the corresponding λ_ext,CORT_ (black, bottom).

To highlight the importance of the subject-to-subject variability, Group 2 was also simulated using the mean value of CORT level across the subjects instead of the times series generated with SMOTE. The explained variability was significantly smaller (*R*^2^ = 0.49 instead of the previous *R*^2^ = 0.59) than the one obtained with the virtual cohort (see Fig L in S1 Text).

#### Combined influence of sleep and CORT

To consider the combined effect of sleep and CORT, we defined

$$\begin{array}{c} \lambda_{\text{ext}}(t)=p_{S}\lambda_{\text{ext},\text{sleep}}(t)+p_{C}\lambda_{\text{ext},\text{CORT}}(t)\;, \end{array}$$ (5)

where λ_ext,sleep_ and λ_ext,CORT_ reflect the hypothesised physiological changes in brain excitability due to sleep and CORT, respectively.

Simulations were carried out over a grid where 0 ≤ *p*_S_ ≤ 1.5 and 0 ≤ *p*_C_ ≤ 1.5. For each parameter combination, we computed the residual sum of squares (*RSS*) to measure the discrepancy between the data and model predictions. More precisely, $RSS=\sum_{i=1}^{24}{\left(y_{i}-{\hat{y}}_{i}\right)}^{2}$, where *y*_*i*_ is the reported ED rate in the *i*^*th*^ 1-hour time interval, while ${\hat{y}}_{i}$ is the model prediction for the corresponding time window.

## Supporting information

S1 Text Supplementary materials. Extended methods and analyses.
(PDF)
