# Supplementary material for: Circadian distribution of epileptiform discharges in epilepsy: Candidate mechanisms of variability
Source: PLoS Comput Biol. 2023 Oct 5;19(10):e1010508. doi: 10.1371/journal.pcbi.1010508 (PMC10581478; doi:10.1371/journal.pcbi.1010508)
Supplement: S1 Text — Extended methods and analyses. (PDF) [file pcbi.1010508.s001.pdf]

# Circadian distribution of epileptiform discharges in epilepsy: candidate mechanisms of variability

Isabella Marinelli*, Jamie J. Walker, Udaya Seneviratne, Wendyl D'Souza, Mark J. Cook, Clare Anderson, Andrew P. Bagshaw, Stafford L. Lightman, Wessel Woldman‡, John R. Terry‡

‡These authors contributed equally to this work.

* i.marinelli@bham.ac.uk

# S1 Text: Supplementary Information

## 1. Clustering and robustness of number of clusters

Potential subgroups are identified using *k-means* clustering, which is executed using the built-in MATLAB function kmeans. In our computation, we repeated the clustering 10 times using new initial cluster centroid positions.

We repeat the clustering using different numbers of clusters (from 1 to 10), and we employ the Calinski-Harabasz criterion (using the MATLAB function evalclusters) to identify the optimal number. This method is based on the Calinski-Harabasz value, which measures the goodness of fit of the data partition: the higher the value, the better that specific number of clusters succeeds in partitioning the data. Moreover, to test the robustness of our choice of considering a 1-hour time window in our study, we modified the bin width of the histograms by defining the total number of bins ($N_{\text{bin}}$) in constructing these histograms. Setting $N_{\text{bin}} = 24$ corresponds to assessing the distribution of the EDs on an hourly basis as presented in the Manuscript.

Fig A shows the Calinski-Harabasz criterion values for each number of clusters tested for different numbers of bins. The highest values are consistently found at two clusters, independent of the specific choice of time window.

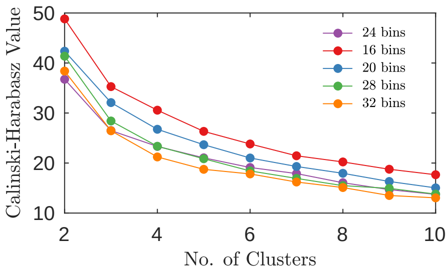


**Fig A. Selecting optimal number of clusters within the IGE cohort.** Calinski-Harabasz values corresponding to different numbers of clusters and time windows.

## 2. Impact of histogram bin width

In the Manuscript, we organized IGE subjects into two clusters based on the similarities in their cumulative epileptiform discharge patterns on an hourly basis. We test the robustness of our result by repeating the clustering for a range of values of $N_{\text{bin}}$ (see Table A and Fig B). The size of these two clusters across the different bin width values is similar, which shows the findings in the main manuscript are robust with respect to the specific choice of bin width.

**Table A. Number of subjects in each group for the different number of bins.**

| No. of Bins | Time Window | Group 1 | Group 2 |
|---|---|---|---|
| 16 | 90 min | 64 | 43 |
| 20 | 72 min | 69 | 38 |
| **24** | **60 min** | **66** | **41** |
| 28 | $\approx$ 51 min | 69 | 38 |
| 32 | 45 min | 60 | 47 |

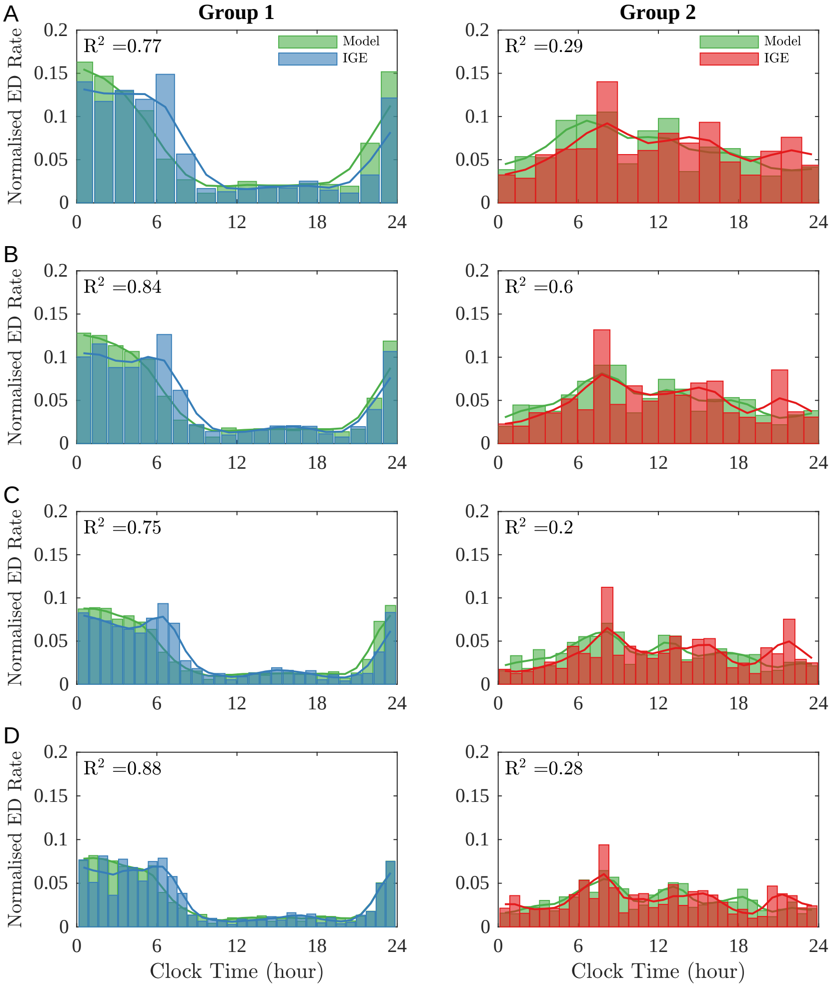


**Fig B. Model results compared with IGE data for different numbers of bins.** Left column: Histogram of epileptiform discharges from Group 1 with IGE (blue) and histogram of epileptiform discharges simulated using the model with $\lambda_{\text{ext}}$ defined to mimic the different brain excitability during sleep stages (green). Right column: Histogram of epileptiform discharges from Group 2 with IGE (red) and histogram of epileptiform discharges simulated using the model with $\lambda_{\text{ext}}$ defined to mimic the impact of cortisol on the brain excitability (green). Histograms were constructed for different numbers of total bins: 16 (A), 20 (B), 28 (C), and 32 (D).

## 3. Clustering when time series are aligned to sleep onset and offset

We can cluster the patients based on the cross-correlation computed when the time series are aligned to the patient's sleep onset and offset. Fig C illustrates the output when the series are aligned to clock time (column A), sleep onset (column B), and sleep offset (column C). Although some patients classified into Group 1 when the clock time is used are now clustered within Group 2 when sleep times are considered (and vice-versa), the overall distributions are robust. Therefore, we decided to use the clock time in our study (see main text).

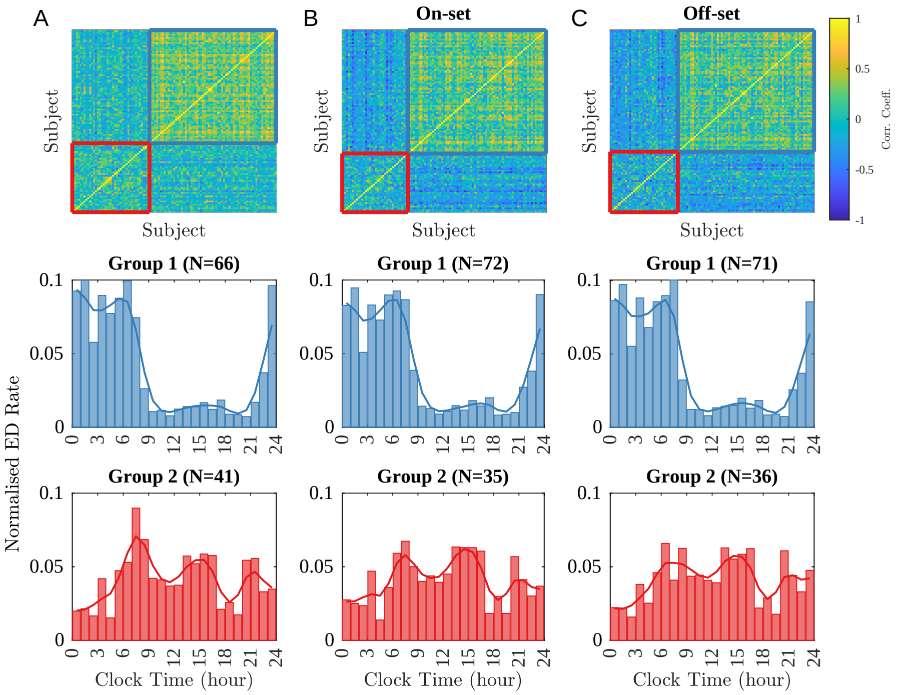


**Fig C. Clustering when the time series are aligned to sleep times.** ED hourly rate patterns were aligned to the clock time (A), sleep onset (B) and sleep offset (C). A pairwise cross-correlation matrix (107 × 107) was calculated to establish similarities within the IGE cohort (first row). The corresponding Group 1 and 2 are shown in the middle and bottom rows, respectively.

## 4. Groups vs type of epilepsy

Table B provides a detailed breakdown of the different syndromes within the two groups. To assess the dependence of the groups on the specific syndrome, we fit a linear model using the MATLAB function fitlm and the model formula

$$\mathsf{Group} \sim \mathsf{Syndrome}.$$

The $p$-value for the test is $p = 0.756$, suggesting there is no strong evidence of an association between the variability in EDs occurrences in the two groups and specific syndromes.

**Table B. Subject characteristics organised by group (Group 1 and Group 2) and syndrome.**

| **Group 1** | | | | |
|---|---|---|---|---|
| Syndrome | Number (%) | Female | Male | Mean Age (min, max) |
| CAE | 11 (17) | 10 | 1 | 22.18 (16,47) |
| JAE | 21 (32) | 11 | 10 | 27.33 (15,45) |
| JME | 18 (27) | 12 | 6 | 31.39 (17, 58) |
| GTCSO | 5 (23) | 9 | 6 | 28.40 (15,42) |
| GGEU | 1 (1) | 1 | 0 | 49 (-) |

| **Group 2** | | | | |
|---|---|---|---|---|
| Syndrome | Number (%) | Female | Male | Mean Age (min, max) |
| CAE | 4 (10) | 1 | 3 | 26 (16,41) |
| JAE | 13 (32) | 8 | 5 | 26.46 (15,48) |
| JME | 10 (24) | 7 | 3 | 27.5 (15,47) |
| GTCSO | 13 (32) | 10 | 3 | 32.54 (17,45) |
| GGEU | 1 (2) | 1 | 0 | 29 (-) |

# 5. Temporal patterns of ED events

To test the dependence of EDs occurrence on the circadian hour time and sleep in Group 1 and 2, we employed a mixed-effect Poisson regression model with the formula:

$$\mathsf{ED} \sim \mathsf{Time} + \mathsf{Sleep} + (1|\mathsf{Subject})$$

where observed variable ED = ($ED_1$, ..., $ED_N$) is a 107×24 matrix where each $ED_i$ corresponds to the ED occurrence for the $i^{th}$ individual of the IGE cohort during the 24-hour time window. The predictor Time represents the circadian time (hours), while Sleep is a 24-element vector whose entrance is 1 if the individual is sleeping and 0 otherwise. Due to the intra-subject variability, we introduce the variable Subject as a random factor. We used the MATLAB function `fitlme` to estimate the slope of the traces corresponding to each measurement.

Table C summarizes the results of the linear regression for Groups 1 and 2. In both groups, there is a statistically significant change in ED counts across time blocks and sleep ($p$-value <0.001). This suggests an impact of sleep on the ED occurrence in Group 2 as well as in Group 1. This result can be explained by noticing that the morning peak in ED events recorded in Group 2 starts during sleep time.

**Table C. Summary of the linear mixed-effects modelling of ED occurrence.** Summary of the linear mixed-effects modelling of ED occurrence in Group 1. Estimates, 95% confidence intervals, and $p$-value (bold, p-value <0.05) for each predictor of ED are shown.

| **Group 1** | | | |
|---|---|---|---|
| Predictors | Estimate | CI | $p$-value |
| (Intercept) | -0.54489 | [-0.9122, -0.1776] | **0.0037** |
| Sleep | 1.451 | [1.3699, 1.5321] | <**0.001** |
| Time | -0.015921 | [-0.0205, -0.0113] | <**0.001** |

| **Group 2** | | | |
|---|---|---|---|
| Predictors | Estimate | CI | $p$-value |
| (Intercept) | 0.32562 | [-0.1615, 0.8127] | 0.1899 |
| Sleep | -0.28703 | [-0.3864, -0.1877] | <**0.001** |
| Time | -0.015982 | [-0.0229, -0.0091] | <**0.001** |

## 6. Examples of other networks

In our study, we used a 4-node network to simulate transitions between a background and a pathological state within a coupled network. Results were similar when smaller (2 and 3-node) or bigger (5-node) networks with different node degrees were used (see Fig D). A larger network size (e.g. $\geq 19$) was not considered, since that would come at a hindering computational cost, and the overall validity of the phenomenological approach is not diminished by the smaller network size.

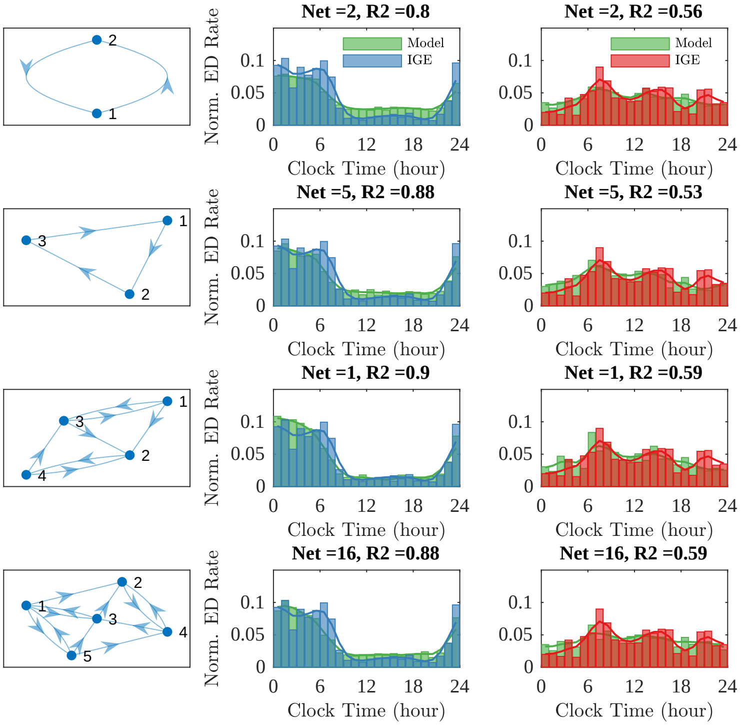


**Fig D. Network selection.** Examples of simulations with different network structures (left column) and the corresponding model simulations for Group 1 (middle column) and Group 2 (right column).

# 7. Null model vs external perturbation

To test whether the model outputs are only due to random noise, we defined the 'null model' as the model with no external input, i.e., $\lambda_{\text{ext}}(t) = 0$. We run this model for both groups and we perform a statistical analysis to check whether there is a statistically significant difference across the day between the null model (`Null`) and the simulation with the external factor (`Sim`). The linear model was fit using the MATLAB function `fitlm` with the formula:

$$\mathsf{Y} \sim \mathsf{Time} + \mathsf{Sleep}$$

where $Y$ is either the null model (no external perturbation) or the model simulation with sleep and CORT only for Group 1 and 2, respectively. Tables D and E summarize the results for the null model and the model accounting for the external factors, respectively.

For both groups, `Sim` shows a statistically relevant change during the day (p-time<0.001), while `Null` shows no changes (p-time>0.1). This result suggests that there is an effect of the external factors on the model simulation and it is not due only to random noise.

**Table D. Summary of the linear modelling of the null model.** Summary of the linear mixed-effects modelling of ED occurrence in Group 1 and 2. Estimates, 95% confidence intervals, and $p$-value (bold, p-value <0.05) for each predictor of ED are shown.

| **Group 1** | | | |
|---|---|---|---|
| Predictors | Estimate | CI | $p$-value |
| (Intercept) | 3.372 | [3.12, 3.62] | <**0.001** |
| Time | -0.003 | [-0.02, 0.01] | 0.64 |
| Sleep | -0.054 | [-0.27, 0.17] | 0.61 |

| **Group 2** | | | |
|---|---|---|---|
| Predictors | Estimate | CI | $p$-value |
| (Intercept) | 3.372 | [3.12, 3.62] | <**0.001** |
| Time | -0.003 | [-0.02, 0.01] | 0.64 |
| Sleep | -0.054 | [-0.27, 0.17] | 0.61 |

**Table E. Summary of the linear modelling of with perturbation.** Summary of the linear modelling of ED occurrence simulated by our model when Sleep is the only external factor in Group 1 and CORT in Group 2. Estimates, 95% confidence intervals, and *p*-value (bold, p-value <0.05) for each predictor of ED are shown.

| **Group 1** | | | |
|---|---|---|---|
| Predictors | Estimate | CI | *p*-value |
| (Intercept) | 6.5324 | [6.3694, 6.6953] | <**0.001** |
| Time | -0.13526 | [-0.14731, -0.12321] | <**0.001** |
| Sleep | -2.403 | [-2.5796, -2.2264] | <**0.001** |

| **Group 2** | | | |
|---|---|---|---|
| Predictors | Estimate | CI | *p*-value |
| (Intercept) | 3.2066 | [2.9783, 3.435] | <**0.001** |
| Time | 0.021373 | [0.0081737, 0.034572] | **0.0029121** |
| Sleep | -0.075574 | [-0.27583, 0.12468] | 0.44133 |

## 8. CORT and sleep mechanisms, inverted

In order to consider all possibilities, we show the model output when CORT only and sleep only are used as mechanisms for Group 1 and 2, respectively (Fig E). As expected, the model performs very poorly with negative $R^2$ in both cases ($R^2$ = -0.15 in Group 1 and $R^2$ = -9.5180 in Group 2), suggesting the model estimation is less accurate than when the mean ED rate is considered.

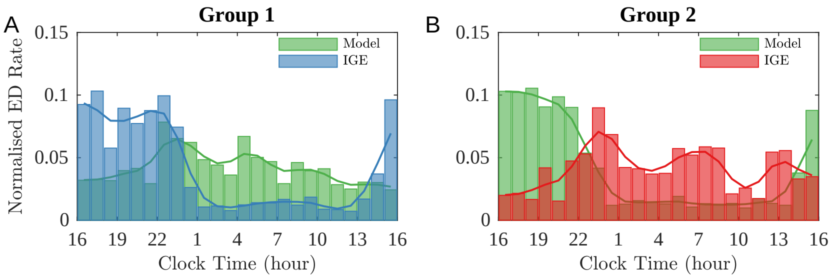


**Fig E. Model results compared with IGE data.** (A) Histogram of EDs from Group 1 with IGE (blue) and histogram of EDs simulated using the model with $\lambda_{\text{ext}}$ defined to mimic the impact of CORT on the brain excitability (green). (B) Histogram of EDs from Group 2 with IGE (red) and histogram of EDs simulated using the model with $\lambda_{\text{ext}}$ defined to mimic the different brain excitability during sleep stages (green).

## 9. $R^2$ statistic for the combined mechanism

To assess the goodness-of-fit of the model, we could also have considered $R^2$, which corresponds to the proportion of variance explained by the model. Fig F shows the values of the $R^2$ statistic computed over a grid of values of $p_S$ and $p_C$

$$R^2 = 1 - \frac{RSS}{TSS} ,$$

where $RSS = \sum_{i=1}^{24} (y_i - \hat{y}_i)^2$ and $TSS = \sum_{i=1}^{24} (y_i - \bar{y})^2$, with $y_i$ the reported ED rate in the $i^{th}$ 1-hour time interval, $\hat{y}_i$ the model prediction for the corresponding time window and $\bar{y}$ the mean value of the observation in the 24-hour period ($\bar{y} = \frac{1}{24} \sum_{i=1}^{24} y_i$).

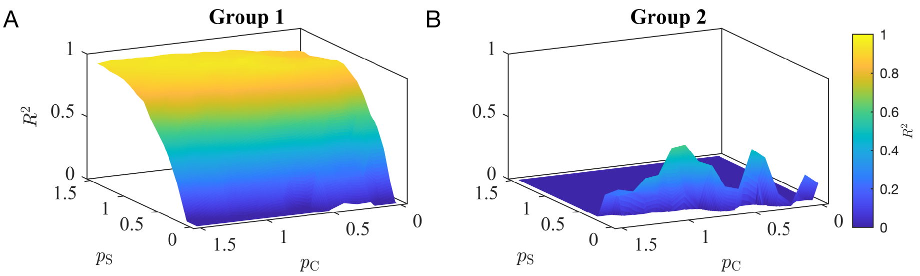


**Fig F. $R^2$ values for the combined mechanisms.** Values of the $R^2$ statistic computed over a grid of values of $p_S$ and $p_C$ for Group 1 (A) and Group 2 (B).

# 10. Robustness of estimating $p_S$ and $p_C$.

In order to assess the robustness of the parameter estimations of $p_S$ and $p_C$, we considered two other approaches: the maximum-likelihood estimation (MLE) and the Monte Carlo Markov Chain (MCMC).

## Maximum-likelihood estimation

The MLE is a method of estimating the parameters of a statistical model given a set of observations by maximising the likelihood function, $\mathcal{L}(\mathbf{X}|\theta)$ (which is effectively equivalent to a cost-function approach), with $\mathbf{X}$ as the vector of observed data and $\theta$ the parameter vector that we want to estimate. In our study, $\mathbf{X}$ is the ED rate and $\theta = (p_S, p_C)$. We define the vector $\mathbf{Y}_\theta$ as the ED rate estimated by our model for a certain value of $\theta$. We then assume that the ED rate $\mathbf{X}$ comes from a normal distribution with mean $\mathbf{Y}_\theta$ and 0.2 standard deviations, that is $\mathbf{X} \sim \mathcal{N}(\mathbf{Y}_\theta, 0.2)$. The value 0.2 corresponds to the mean of the standard deviation values computed hourly across the cohort.

We therefore compute the values of $\mathcal{L}(\mathbf{X}|p_S, p_C)$ over a grid where $0 \leq p_S \leq 1.5$ and $0 \leq p_C \leq 1.5$. Fig G illustrates the likelihood values for Group 1 (panel A) and Group 2 (panel B). We find that the likelihood function is maximal when $p_S = 1.1$ and $p_C = 0.6$ for Group 1 and $p_S = 0$ and $p_C = 1$ for Group 2. This result is consistent with what has been found using the $RSS$ approach in the main text.

## Monte Carlo Markov Chain

An alternative method to estimate $p_S$ and $p_C$ is the MCMC. In our study, we implemented an adaptive Metropolis (AM) algorithm to estimate the parameters $\theta = (p_S, p_C)$ as described in [1, 2].

As with the MLE, we assume $\mathbf{X} \sim \mathcal{N}(\mathbf{Y}_\theta, 0.2)$ and we define the prior distribution for the parameter $\theta = (p_S, p_C)$ as the truncated multivariate

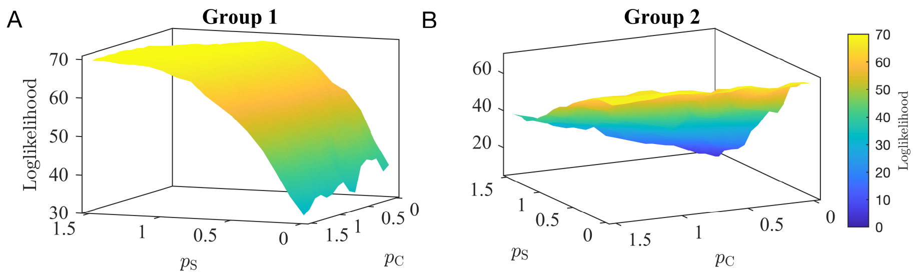


**Fig G. Likelihood function values for the combined mechanism.** Values of the likelihood function ($\mathcal{L}(\mathbf{X}|p_S, p_C)$) computed over a grid at intervals of 0.1 of values of $p_S$ and $p_C$ for Group 1 (A) and Group 2 (B).

normal distribution $\pi := \mathcal{N}_{tr}([1,0],\Sigma_0)$ for Group 1 and $\pi := \mathcal{N}_{tr}([0,1],\Sigma_0)$ for Group 2, where the covariance matrix $\Sigma_0$ is defined in Eq. 1.

$$\Sigma_i := (1-\beta)^2(2.38)^2\frac{\Sigma_{i-1}}{d} + \beta^2(0.1)^2\frac{I_d}{d}, \tag{1}$$

where $\beta = 1$ if $i \leq 4$ and $\beta = 0.05$ otherwise (see [2] for more details).

In short, at every sampler iteration $i$, we suggest a proposal distribution $\bar{\pi} := \mathcal{N}_{tr}(\theta^{i-1}, \Sigma_i)$, where $\theta^{i-1}$ is the latest accepted value and $\Sigma_i$ is the covariance matrix defined in Eq. 1, and we extract the proposed parameter $\theta^i$ from $\bar{\pi}$. We then compute the probability $\alpha := \min\left(1, \frac{\alpha^i}{\alpha^{i-1}}\right)$, with $\alpha^i = \mathcal{L}(X|\theta^i)\pi(\theta^i)\bar{\pi}(\theta^i)$. If $\alpha > u$ (with $u \sim \mathcal{U}([0,1])$), the parameter is accepted and the mean and the covariance matrix of the proposal distribution are updated, that is $\theta^{i+1} \sim \mathcal{N}_{tr}(\theta^i, \Sigma_{i+1})$. Otherwise, the proposed parameter is rejected and $\theta^{i+1} \sim \mathcal{N}_{tr}(\theta^{i-1}, \Sigma_{i+1})$. The result is a time series of accepted proposed parameters $\{\theta\}_i$ (posterior distribution).

Fig H shows the time series for four different initial values (sampled from the prior distribution $\pi$) of the parameters $p_S$ and $p_C$ for both Group 1 and Group 2 (top row) and the corresponding auto-correlation values (bottom row). We ran the MCMC for 5 thousand iterations of which the first one thousand were discarded as burn-in. From the remaining 4 thousand, we recorded samples every 4 iterations for a total of one thousand.

The final values are given by the median of $\{\theta\}_i$ and are reported in Table F. Although the single values are not identical given the different approaches (Bayesian vs Frequentist), the parameters estimated with the $RSS$ and MLE approaches are within the 95% confidence interval estimated with MCMC. More precisely, sleep is shown to be the main driver in Group 1 ($p_S > 1$), although there is a non-negligible CORT component ($p_C \sim 0.3$). Conversely, in Group 2, sleep is not playing a role in shaping the ED distribution ($p_C \sim 0$) while CORT is the main driver ($p_C > 1$).

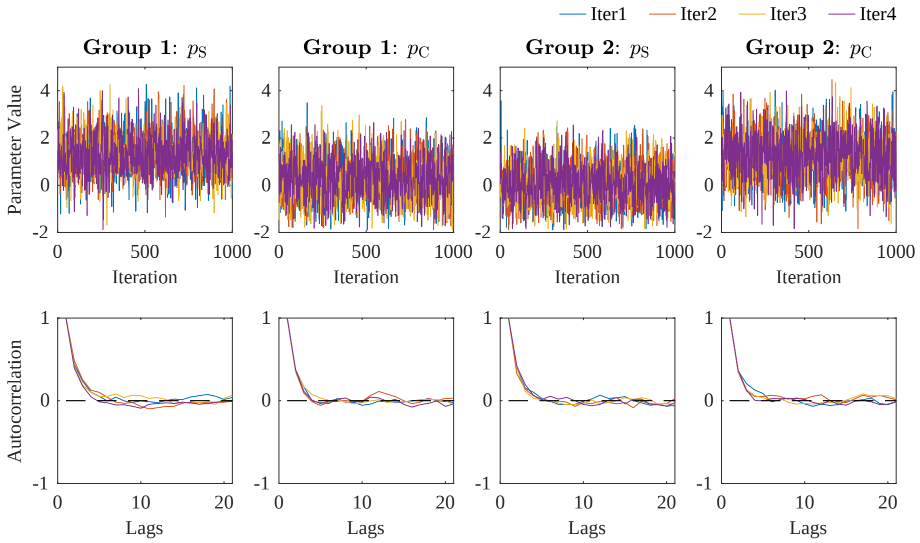


**Fig H. Results for the MCMC algorithm.** Time series of the parameter values obtained with the adaptive Metropolis for the two parameters $p_S$ and $p_C$ for both Group 1 and Group 2 (top row) and the corresponding autocorrelation function (bottom row). The different colours correspond to the different initialization of the parameters.

**Table F. Parameter estimation with MCMC.** Summary of the estimations for the parameters $p_S$ and $p_C$ obtained with the AM MCMC for each initial condition and each of the two groups, and the corresponding 95% confidence interval identified by the first and third quartiles (2.5% percentile and 97.5% percentile, respectively) of the posterior distribution.

| **Group 1** | | |
|---|---|---|
| Iteration | $p_S$ (95%CI) | $p_C$ (95%CI) |
| 1 | 1.27 (0.71, 3.12) | 0.26 (-0.31, 2.20) |
| 2 | 1.26 (0.80, 3.05) | 0.24 (-0.34, 1.93) |
| 3 | 1.29 (0.78, 3.09) | 0.30 (-0.31, 2.09) |
| 4 | 1.28 (0.75, 3.00) | 0.32 (-0.23, 2.06) |

| **Group 2** | | |
|---|---|---|
| Iteration | $p_S$ (95%CI) | $p_C$ (95%CI) |
| 1 | 0.04 (-0.42, 1.70) | 1.25 (0.62, 3.10) |
| 2 | -0.03 (-0.53, 1.76) | 1.20 (0.60, 3.16) |
| 3 | 0.03 (-0.48, 1.91) | 1.20 (0.57, 3.13) |
| 4 | 0.06 (-0.43, 1.71) | 1.19 (0.60, 3.16) |

## 11. Subgroups

We can further consider clustering patients within each group based on the similarity in their pattern of EDs. In both groups, we can identify two sub-groups (Fig I).

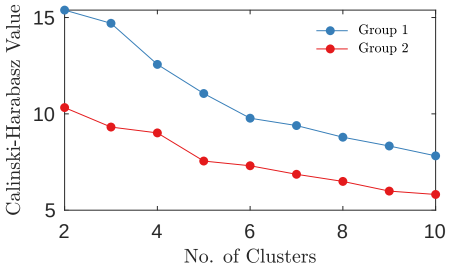


**Fig I. Selecting optimal number of clusters within the two groups.** Calinski-Harabasz values corresponding to Group 1 (blue) and Group (red).

Fig J illustrates the subsequent subgroups. Interestingly, this analysis identifies the subjects in Group 2 responsible for the evening peak, which is unlikely to be explained by the CORT dynamics.

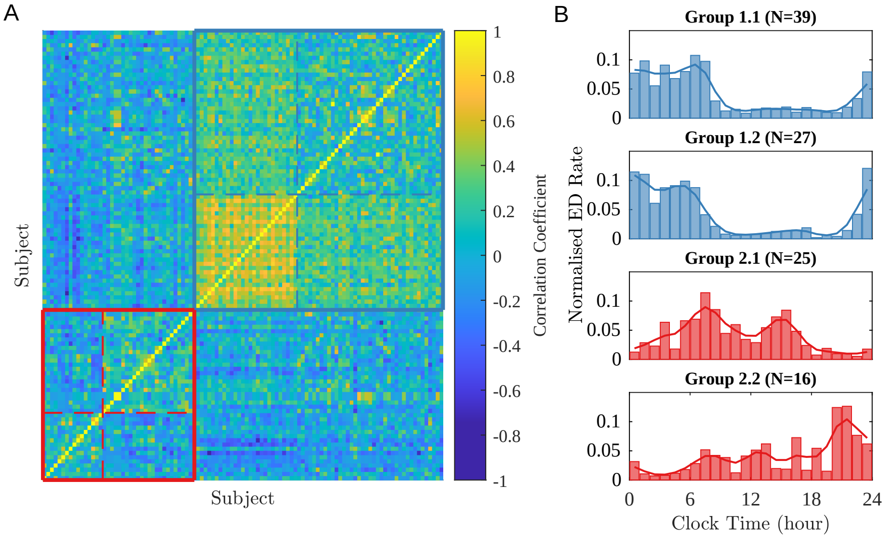


**Fig J. Subgroups.** (A) Pairwise cross-correlation matrix (of size $107 \times 107$) divided by sub-clusters. (B) Group 1 sub-groups (blue): Group 1.1 (N = 39) and Group 1.2 (N = 27). Group 2 sub-groups (red): Group 2.1 (N = 25) and Group 2.2 (N = 16).

Fig K illustrates the optimal fit with the corresponding value of $p_S$, $p_C$, and $R^2$.

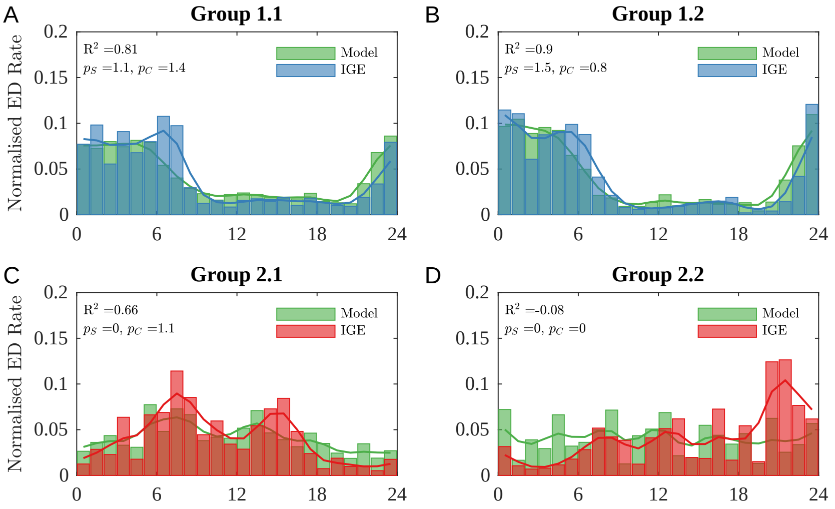


**Fig K. Best model fit for the subgroups compared with IGE data.** Histograms of epileptiform discharges from the subgroups Group 1.1 (blue, A), 1.2 (blue, B), 2.1 (red, C) and 2.2 (red, D) and histogram of epileptiform discharges simulated using the model with $p_{\mathrm{S}}\lambda_{\mathrm{ext,sleep}} + p_{\mathrm{C}}\lambda_{\mathrm{ext,CORT}}$ defined to mimic the external factors on the brain excitability (green). The values of $p_{\mathrm{S}}$, $p_{\mathrm{C}}$, and $R^2$ are shown in the corresponding panel.

## 12. The value of subject-specific CORT levels

A mixed-effect Poisson regression model was implemented to investigate the differences in cortisol levels across the subject:

$$\mathsf{CORT} \sim \mathsf{Subject} + (1|\mathsf{Time})$$

We found that there is a statistically significant inter-subject variability ($p < 0.001$). Table G illustrates the analysis output.

**Table G. Summary of the linear mixed-effects modelling of CORT levels.** Summary of the linear mixed-effects modelling of CORT levels. Estimates, 95% confidence intervals, and $p$-value (bold, p-value <0.05) for each predictor of ED are shown.

| Predictors | Estimate | CI | $p$-value |
|---|---|---|---|
| (Intercept) | 5.7533 | [4.9263, 6.5803] | <**0.001** |
| Subject | 0.45488 | [0.29195, 0.6178] | <**0.001** |

To highlight the importance of the CORT subject-to-subject variability, Group 2 was also simulated using the mean value of CORT level across the subjects (Panel A in Fig L) instead of the times series generated with SOTE. The model output is shown in Panel B of Fig L and accounts for 50% of the variability ($R^2 = 0.49$), which is about 10% less than the one obtained with the virtual cohort.

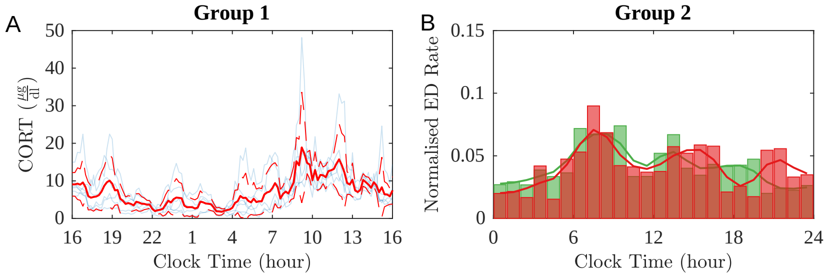


**Fig L. Model result compared with IGE data for Group 2.** (A) CORT mean (solid red line) across the individuals (blue lines). The dashed lines indicate the 95% confidence interval. (B) Histogram of epileptiform discharges from Group 2 with IGE (blue) and histogram of epileptiform discharges simulated using the CORT mean value to model $\lambda_{\text{ext,CORT}}$ (green).

## 13. Representative model simulation

The mathematical model used in this study is based on the normal form of a subcritical Hopf bifurcation:

$$\dot{z_i} = (\lambda_i - 1 + i\omega)z_i + 2z_i|z_i|^2 - z_i|z_i|^4 + \beta \sum_{j=1}^{N} A_{ij}(z_j - z_i) + \alpha \mathrm{d}W(t) \ , \quad (2)$$

$$\dot{\lambda_i} = \frac{1}{\tau}\left[\lambda_{\mathrm{base}} + (\lambda_{\mathrm{ext}})_i - \lambda_i - |z_i|^2\right] \ , \quad (3)$$

where $z_i(t)$ is dynamics of the $i^{th}$ node (with $i = 1, ..., N$), $W(t)$ is a complex Wiener process, $\lambda(t)$ is the excitability of node $i$, $\lambda_{\mathrm{base}}$ the baseline level of excitability, $\lambda_{\mathrm{ext}}(t)$ the external perturbations to the excitability, $\omega$ determines the oscillation frequency and $\alpha$ determines the level of noise for the complex Wiener process $W(t)$. Typical parameter values for the model are given in Table 2 in the main text, whilst $A$ is an adjacency matrix, i.e. $A_{i,j}$ is 1 if there is a connection between the $i^{th}$ and $j^{th}$ regions and 0 otherwise. For simplicity, all simulations were performed with a directed and connected 4-node graph ($N = 4$) (Fig 9 in the main text). Numerical simulations were obtained using an Euler-Maruyama scheme to find approximate solutions to the system of stochastic differential equations (SDEs) with $dt = 10^{-3}$. The method was implemented in MATLAB R2021a (MathWorks Inc., Natick, MA) to simulate 24-hour brain activity.

Fig M illustrates representative model simulation: the variables $z_i$ and $\lambda_i$ are shown in Panels (A) and (B), respectively, while the $\lambda_{\mathrm{ext}}(t)$ employed in the model is given by the sum of $\lambda_{\mathrm{ext,sleep}}$ and $\lambda_{\mathrm{ext,CORT}}$ shown in Panel (C). The values of the corresponding ED rate are presented in Panel (D).

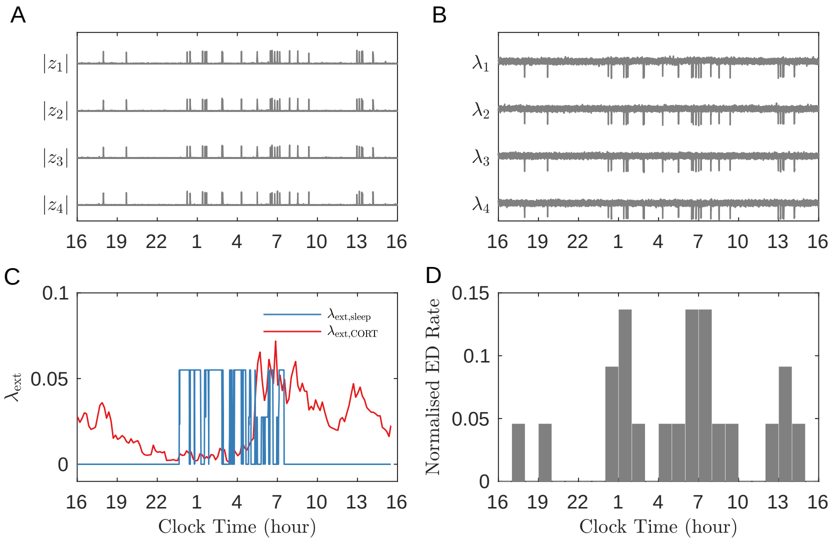


**Fig M. Simulation representative.** Numerical simulations of the variable $z_i$ (A) and $\lambda_i$ (B), with $i = 1, ..., 4$. The $\lambda_{\text{ext}}$ used in the simulation is given by the sum of $\lambda_{\text{ext,sleep}}$ and $\lambda_{\text{ext,CORT}}$ shown in (C). The resulting ED rate histogram is reported in (D).

# Bibliography

[1] Haario H, Saksman E, Tamminen J. An adaptive Metropolis algorithm. Bernoulli. 2001;7. doi:10.2307/3318737.

[2] Roberts GO, Rosenthal JS. Examples of Adaptive MCMC. https://doiorg/101198/jcgs200906134. 2012;18:349–367. doi:10.1198/JCGS.2009.06134.
